# Intrarenal arteriosclerosis and telomere attrition associate with dysregulation of the cholesterol pathway

**DOI:** 10.18632/aging.103098

**Published:** 2020-04-30

**Authors:** Katrien De Vusser, Ellen Winckelmans, Dries Martens, Evelyne Lerut, Dirk Kuypers, Tim Nawrot, Maarten Naesens

**Affiliations:** 1 Department of Microbiology and Immunology, KU Leuven – University of Leuven, Leuven, Belgium; 2 Department of Nephrology and Renal Transplantation, University Hospitals Leuven, Leuven, Belgium; 3 Centre for Environmental Sciences, Hasselt University, Hasselt, Belgium; 4 Department of Imaging and Pathology, KU Leuven – University of Leuven, Leuven, Belgium; 5 Department of Pathology, University Hospitals Leuven, Leuven, Belgium; 6 Department of Public Health and Primary Care, KU Leuven – University of Leuven, Leuven, Belgium

**Keywords:** aging, senescence, telomeres, cholesterol pathway, arteriosclerosis

## Abstract

Background: Recently, we demonstrated that arteriosclerosis in the smaller intrarenal arteries is associated with shorter telomere length, independently of history of cardiovascular events and calendar age. This suggests that intrarenal arteriosclerosis reflects replicative senescence, although the underlying molecular alterations remain unclear.

Results: Shorter intrarenal telomere length associated significantly with the presence of renal arteriosclerosis (T/S ratio 0.91±0.15 vs. 1.20±0.23 with vs. without arteriosclerosis, p=0.007, test cohort; T/S ratio 0.98 ±0.26 vs. 1.03 ±0.18 with vs. without arteriosclerosis, p=0.02, validation cohort). The presence versus absence of intrarenal arteriosclerosis was associated with differential expression of 1472 transcripts. Pathway analysis revealed enrichment of molecules involved in the superpathway of cholesterol biosynthesis as the most significant. The differential expression of these genes was confirmed in the independent validation cohort. Furthermore, the specific mRNA expression of the molecules in the superpathway of cholesterol biosynthesis associated significantly with intrarenal telomere length, and with history of cardiovascular events.

Interpretation: Our study illustrates that the superpathway of cholesterol biosynthesis interacts with the previously published association between shorter telomere length and arteriosclerosis.

Methods: This study included a test cohort of 40 consecutive kidney donors (calendar age 48.0 ± 15), with biopsies obtained prior to transplantation. Intrarenal leucocyte telomere length content was assessed using quantitative RT-PCR. Whole genome microarray mRNA expression analysis was performed using Affymetrix Gene 2.0 ST arrays. We investigated the associations between mRNA gene expression, telomere length as marker of replicative senescence, and intrarenal arteriosclerosis (Banff “cv” score = vascular fibrous intimal thickening = intimal hyperplasia) using adjusted multiple regression models. For biological interpretation and pathway overrepresentation analysis, we used Ingenuity Pathway Analysis. The significant pathways and genes were validated in an independent validation cohort of 173 kidney biopsies obtained prior to transplantation.

## INTRODUCTION

Telomeres are complexes of tandem TTAGGG repeats of 5000 to 15000 base pairs that reside at the ends of chromosomes [1]. Their main function is to cap these chromosome ends and prevent chromosomal instability [2]. Telomeres shorten by each cell division, until a critical length is reached. This leads to permanent and irreversible growth arrest, referred to as replicative senescence [3]. Telomere length is a well-established marker of biological age [4]. Although telomere length is partly heritable, there are major differences in telomere length even among monozygotic twins, which illustrates that environmental factors are important in telomere attrition rate [5].

Recently, we illustrated and validated that arteriosclerosis in smaller intrarenal arteries of kidneys is associated with shorter telomere length, suggesting a role of telomere shortening or biological aging in the development renal arteriosclerosis. Moreover, we described that shorter (intrarenal) telomere length associates with history of hypertension and cardiovascular events in a cohort of native kidneys used for transplantation [6]. This association between shorter telomere length and clinical cardiovascular disease has also been described in the cardiovascular field [7–9]. It could therefore be hypothesized that renal arteriosclerosis reflects a specific senescence process. In contrast, other lesions included in the phenotype of “nephrosclerosis” (glomerulosclerosis, interstitial fibrosis, tubular atrophy) did not associate with history of cardiovascular events, hypertension or telomere length, and likely represent cumulative non-specific injury processes, rather than specific aging processes [6].

The lesions of arteriosclerosis begin as the intima of the arterial wall fills up with the deposition of cellular waste and ends with a thickening and loss of elasticity of the arterial walls [10, 11]. Different molecular pathways like cell proliferation regulatory pathways including genes involved in the cell cycle regulation checkpoints, cytokine-associated signaling pathways and lipoprotein pathways have been associated with the presence of arteriosclerosis [12–14].

Given the recent suggestions that intrarenal arteriosclerosis associates with replicative senescence, but the lack of molecular underpinning of these findings, we investigated the association between intrarenal telomere length and intrarenal arteriosclerosis at the molecular level using micro-array gene expression analyses.

## RESULTS

### Population characteristics

Between February 2013 and April 2015, 297 renal transplantations were performed at the University Hospitals Leuven, of which 213 had a pre-implantation renal allograft biopsy for evaluation of telomere length, gene expression and histological evaluation available (40 test cohort, 173 validation cohort). In the test cohort mean T/S ratio of intrarenal telomere length was 1.17±0.20 (range 0.69-1.74). In the validation cohort mean T/S ratio of intrarenal telomere length was 1.02±0.19 (range 0.59-1.74). Table 1 summarizes the characteristics of our cohort and the histology of the biopsies that were included. Shorter intrarenal telomere length associated significantly with the presence of renal arteriosclerosis in the test cohort (T/S ratio 0.91±0.15 vs. 1.20±0.23 with vs. without arteriosclerosis; p=0.007) and in the validation cohort (T/S ratio 0.98 ±0.26 vs. 1.03 ±0.18 with vs. without arteriosclerosis, p=0.02).

**Table 1:** Demographics and histology of the subjects and biopsies included in this study.

**Demographics**	***Test cohort***	***Validation cohort***
N	*40*	*173*
**Donor Characteristics**		
Calendar Age (years)	48.0 ± 15	48.7 ± 15
Male Gender % (N)	57.5% [43]	49.1% (85)
Living/Deceased Donor % (N)	10.0% (4)/90.0% (36)	11.6% (20)/88.4% (153)
Brain Death / Cardiac Death % (N)	72.2%(26)/27.8%(10)	79.0%(120)/21.0%(32)
Body Mass Index (kg/m^2^)	29.0 ± 5.3	28.5 ± 11.9
History of Cardiovascular Events % (N)	40.0% (16)	43.4% [44]
Cold Ischemia Time (hours)	12.0 ± 5.8	11.5 ± 5.9
**Histological Characteristics**		
Intrarenal telomere length (T/S ratio)	1.17±0.20	1.02±0.19
Banff Arteriosclerosis grade % (N) (159)	0= 87.5% [45] 1-2= 12.5% (5)	0= 87.4% (139) 1-2= 12.6% (20)

Data are expressed as mean ± standard deviation unless otherwise specified; ^#^eGFR was calculated using the MDRD formula [15, 42].

### Micro-array gene expression and intrarenal telomere length

**Figure 1:**
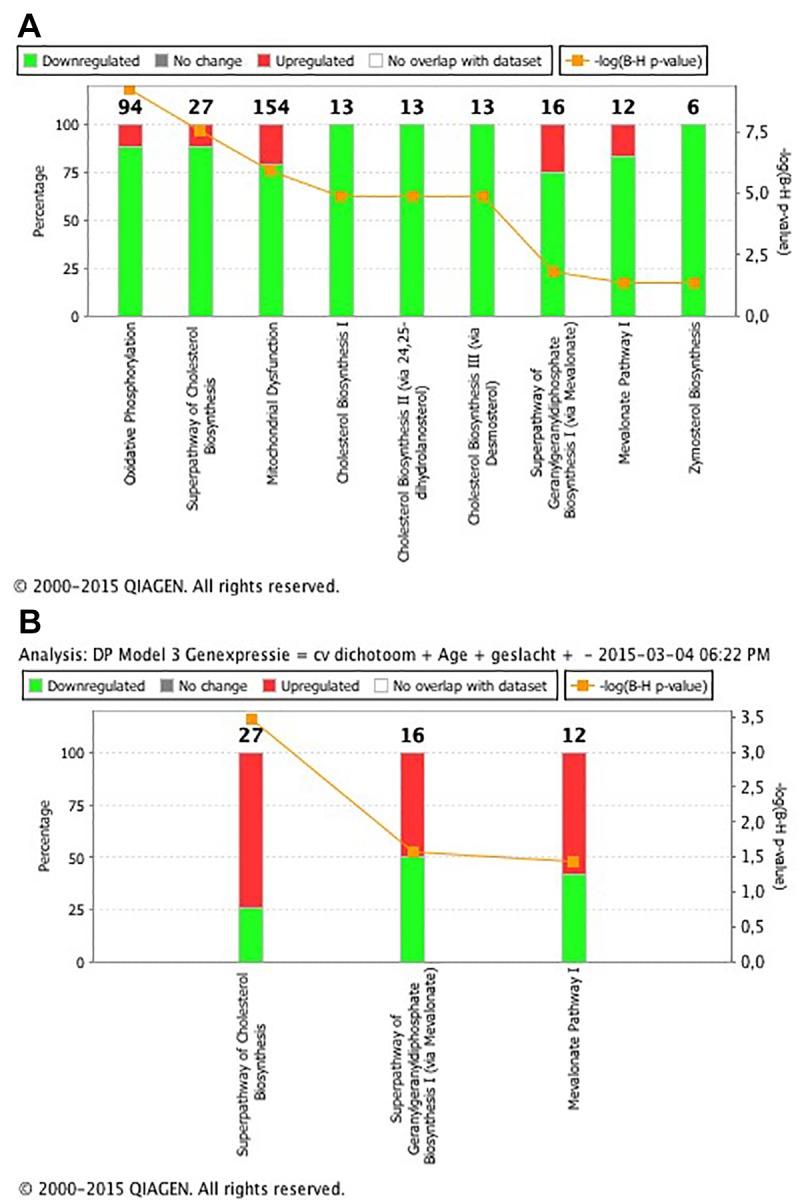
(**A**) List of the 9 significant canonical pathways associated with telomere length. The significance of the pathways is expressed as the Benjamini-Hochberg-adjusted p value (q value), which is corrected for multiple testing. The percentage on the left y axis represents the percentage of over- and lower expressed genes in the pathway. The numerical value at the top of each bar represents the total number of genes in the canonical pathway. Pathway analysis revealed enrichment of transcripts coding for proteins of the oxidative phosphorylation pathway (q=5.75x10^-10^), transcripts involved in the superpathway of cholesterol biosynthesis (q=2.96x10^-8^) and transcripts involved in the mitochondrial dysfunction pathway (q=1.26x0^-6^) as the first three most significant pathways. (**B**) List of the 3 significant canonical pathways associated with intrarenal arteriosclerosis. The significance of the pathways is expressed as the Benjamini-Hochberg-adjusted p value (q value), which is corrected for multiple testing. The percentage on the left y axis represents the over- and lower expressed genes in the pathway. The numerical value at the top of each bar represents the total number of genes in the canonical pathway. Pathway analysis revealed enrichment of transcripts coding for proteins of the superpathway of cholesterol biosynthesis (q=0.0003), transcripts involved in the superpathway of geranylgeranyldiphosphate biosynthesis I (via mevalonate) (q=0.02) and transcripts involved in the mevalonate pathway I (q=0.03) as the three significant pathways.

In total, expression of 1300 transcripts significantly associated with intrarenal telomere length at p<0.05 in multivariable linear regression analysis (adjusted for calendar age, gender and batch), of which 629 were significantly higher expressed in samples with shorter telomeres. These individual transcripts did not pass Benjamini-Hochberg FDR adjustment. Pathway analysis of the 1300 transcripts revealed enrichment of genes coding for proteins of the oxidative phosphorylation pathway (q=5.75x10 [-10]), genes involved in the superpathway of cholesterol biosynthesis (q=2.96x10^-8^) and genes involved in the mitochondrial dysfunction pathway (q=1.26x0^-6^) as the top three significant pathways (Figure 1A and Supplementary Table 1. The 36 genes that were implicated in these pathways all had increased expression with telomere shortening (Supplementary Table 2).

### Micro-array gene expression and intrarenal arteriosclerosis

In total, 1472 transcripts significantly associated with intrarenal arteriosclerosis (adjusted for calendar age, gender and batch), of which 674 genes had significantly increased expression in biopsies with arteriosclerosis. These individual transcripts did not pass Benjamini-Hochberg FDR adjustment. Pathway analysis revealed enrichment of genes coding for proteins of the superpathway of cholesterol biosynthesis (q=0.0003), genes involved in the superpathway of geranylgeranyldiphosphate biosynthesis I (via mevalonate) (q=0.02) and genes involved in the mevalonate pathway I (q=0.03) as the three top significant pathways (Figure 1B and Supplementary Table 1). In these pathways, 14 genes were significantly associated with presence of arteriosclerosis; 12 overexpressed and 2 lower expressed in biopsies with arteriosclerosis (Supplementary Table 3).

### Validation of the superpathway of cholesterol biosynthesis

**Figure 2:**
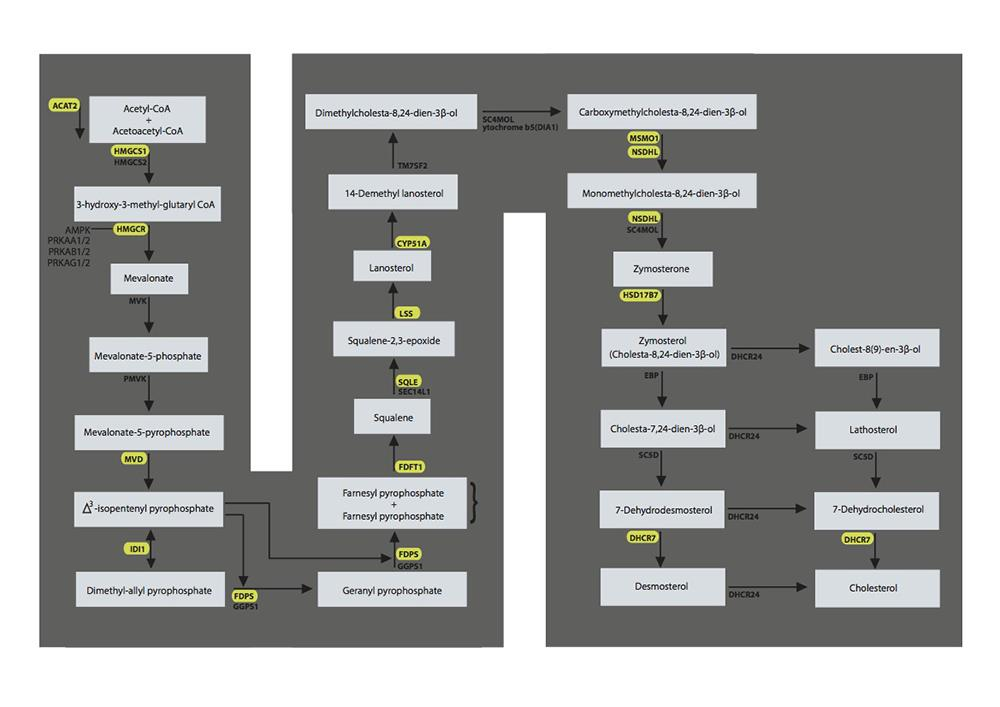
**The superpathway of cholesterol biosynthesis with the genes significant in the test cohort and validated in the validation cohort marked in yellow.** LSS = lanosterol synthase, FDPS = farnesyl diphosphate synthase; DHCR7 =7-dehydrocholesterol reductase; HMGCR = 3-hydroxy-3-methylglutaryl-CoA reductase; FDFT1 = farnesyl-diphosphate farnesyltransferase 1; IDI1 = isopentenyl-diphosphate delta isomerase 1; ACAT2 = acetyl-CoA acetyltransferase 2; NSDHL = NAD(P) dependent steroid dehydrogenase-like; HMGCS1 = 3-hydroxy-3-methylglutaryl-CoA synthase 1; CYP51A1 =cytochrome P450, family 51, subfamily A, polypeptide 1; SQLE = squalene epoxidase; MSMO1 =methylsterol monooxygenase 1; MVD = mevalonate (diphospho) decarboxylase; HSD17B7 = hydroxysteroid (17-beta) dehydrogenase 7.

**Figure 3:**
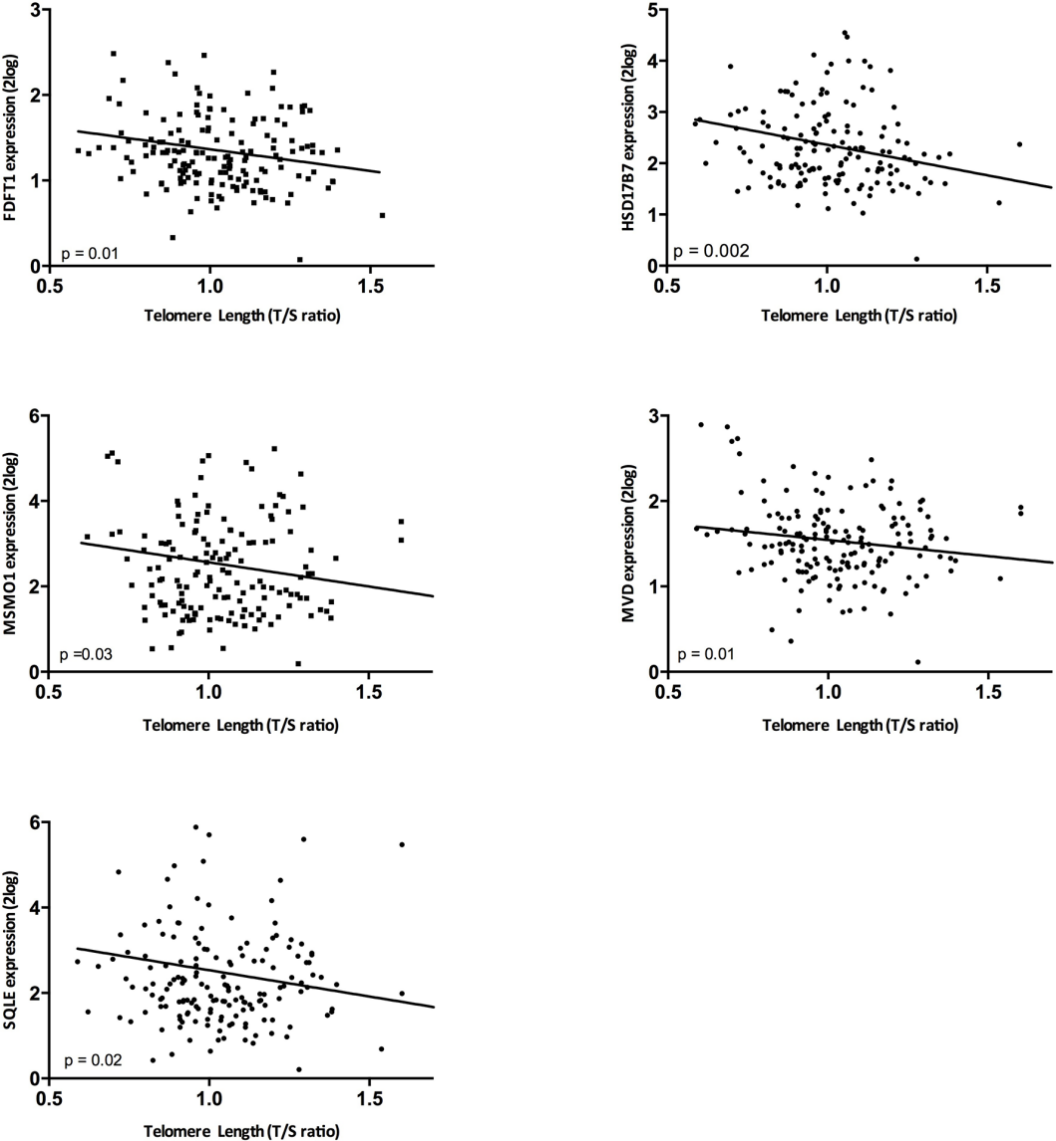
**The correlation between telomere length and gene expression in the validation cohort.** Associations between telomere length and gene expression were assessed by means of Spearman correlations.

Given the significant enrichment of the superpathway of cholesterol biosynthesis in both the analysis of telomere length and intrarenal arteriosclerosis, in these unrelated analyses (Supplementary Table 1), we validated selected genes of this pathway (Table 1; Figure 2 and Supplementary Tables 2, 4) in the independent validation cohort (N=159). In 14 biopsies (8%) there was no evaluation of arteriosclerosis because there was no vessel present in the biopsy. Telomere length correlated significantly with the expression of FDFT1 (p= 0.01, r=-0.20), MSMO1 (p= 0.03, r=-0.16), MVD (p= 0.01, r=-0.16), HSD17B7 (p= 0.002, r=-0.24) and SQLE (p= 0.02, r=-0.17) (Figure 3). When the genes involved in this pathway were expressed as the first principal component of the whole pathway, also this parameter correlated highly significantly with intrarenal telomere length (p= 0.002).

**Figure 4:**
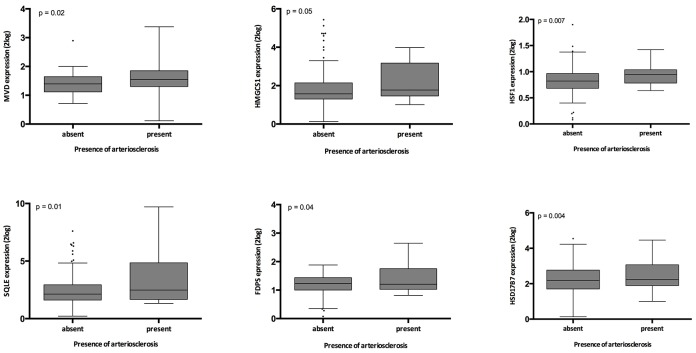
**Relation between intrarenal arteriosclerosis and gene expression in the validation cohort.** The p-values represent non-parametric ANOVA. The horizontal lines within the boxes indicate means, the upper and lower ends of the boxes indicate standard deviations, and the whiskers indicate 95^th^ percentiles.

Presence of arteriosclerosis associated significantly with expression of SQLE, FDPS, MVD, HMGCS1, HSD17B7 and HSF1 (all p≤0.05) (Supplementary Table 4, Figure 4). When the genes involved in this pathway were expressed as the first principal component of the whole pathway, also this parameter associated highly significantly with intrarenal arteriosclerosis (p= 0.006).

### The superpathway of cholesterol biosynthesis and history of cardiovascular events

**Figure 5:**
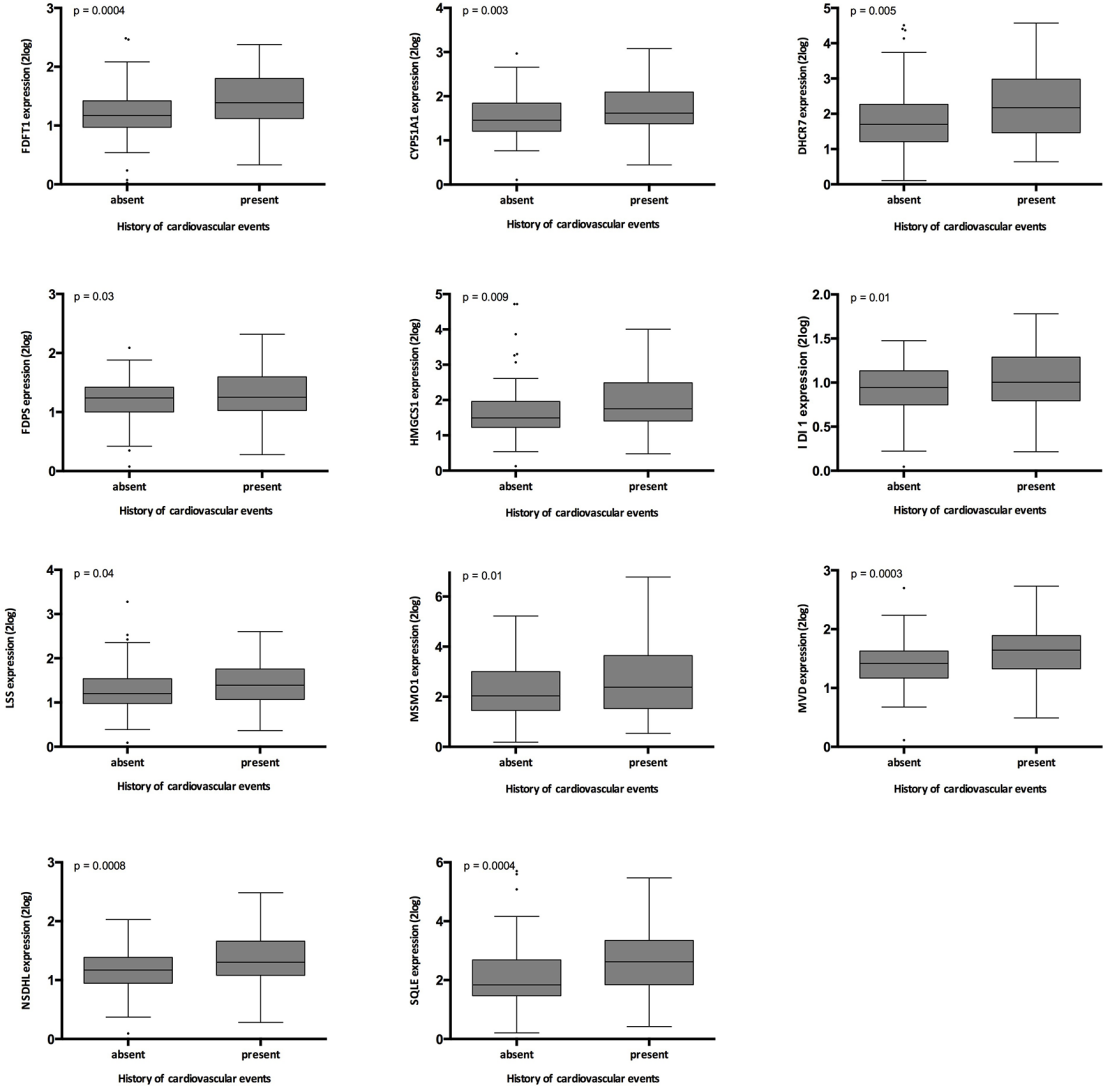
**Relation between history of cardiovascular events in the validation cohort and gene expression.** The p-values represent non-parametric ANOVA. The horizontal lines within the boxes indicate means, the upper and lower ends of the boxes indicate standard deviations, and the whiskers indicate 95^th^ percentiles.

History of cardiovascular events associated significantly with the expression of FDFT; DHCR7; FDPS; HMGCS1; IDI1; LSS; MSMO1; MVD; NSDHL; SQLE; CYP51A1 (Supplementary Table 4, and Figure 5). When the superpathway of cholesterol biosynthesis was expressed as the first principal component of all genes involved in this pathway, this parameter associated highly significantly with history of cardiovascular events (p= 0.004).

## DISCUSSION

In the current study, whole genome expression analyses in kidney biopsies revealed significant enrichment of the superpathway of cholesterol biosynthesis in the gene expression changes that associate with telomere attrition and with intrarenal arteriosclerosis. The most relevant genes of this pathway were confirmed in an independent validation cohort. The expression of selected genes of the cholesterol pathway also associated with history of cardiovascular events.

The superpathway of cholesterol biosynthesis summarizes the different routes of cholesterol biosynthesis. Less than half of the cholesterol in the body derives from biosynthesis *de novo*. Cholesterol biosynthesis in the liver accounts for approximately 10% and in the intestine approximately 15% of the daily production, but all human cells can produce cholesterol. There is evidence of the expression of cholesterol pathways and production of cholesterol by the kidney, more specifically by the proximal tubular cells, mesangial cells and podocytes [15–17]. Moreover, there is endogenous production of cholesterol by vascular cells, which are also present in the kidney [18]. Synthesis of cholesterol originates from the transport of acetyl-CoA from the mitochondria to the cytosol. The mevalonate pathway of cholesterol synthesis initiates with formation of 3-hydroxy-3-methylglutaryl-coenzyme A (HMG-CoA) from acetyl-coenzyme A, through enzymatic interaction by HMG-coenzyme A synthase (HMGCS). HMGCS expression was increased in our analysis, both in the test and in the validation cohort. The rate-limiting step in cholesterol synthesis occurs at HMG-CoA reductase (HMGCR), which was also overexpressed in our micro-array analysis in the presence of telomere attrition and presence of arteriosclerosis, although we could not validate this in the RT-PCR experiment. The synthesis of squalene represents the first cholesterol-specific step in the cholesterol synthesis pathway, and several transcripts in this specific pathway were overexpressed in our analyses, like squalene epoxidase (SQLE), farnesyl diphosphate synthase (FDPS), and transferase (FDFT1). Squalene then undergoes a two-step cyclization to yield lanosterol, which is finally converted to cholesterol, depending on a large chain of intermediary products (Figure 2). Several of the key enzymes in this downstream cascade were differentially expressed in our analyses.

Intracellular lipid content is importantly influenced by the rate of uptake of lipids from the circulation, and by this from circulating lipid levels [19]. In patients with homozygous familial hypercholesterolemia, the superpathway of cholesterol synthesis is upregulated because of defective binding of lipoproteins to cells [20, 21]. Intracellular lipid synthesis is regulated by sterol regulatory element binding proteins (SREBP), and intracellular cholesterol content is heavily regulated to prevent toxicity from progressive intracellular accumulation of cholesterol [22]. Competitive inhibitors of HMG-CoA Reductase (HMGCR), better known as statins, inhibit cholesterol synthesis and diminish cardiovascular risk by inhibition of the first step of this pathway. The contribution of lipid metabolism to atherosclerosis has been reviewed previously [19, 23, 24]. Intracellular lipid accumulation is proatherogenic through an array of secondary effects, including immune cell stimulation and endothelial cell dysfunction [19]. Moreover, transformation of contractile smooth muscle cells to migratory, proliferative and synthetic smooth muscle cells has been associated with alterations of cellular lipid homeostasis and overexpression of genes involved in cholesterol biosynthesis [25], although it should be emphasized that these last data need further validation. Moreover, homocysteine leads to dysregulated cholesterol synthesis in endothelial cells, which could be involved In the proatherogenic effects of hyperhomocysteinemia [26].

Intrarenal lipid metabolism was investigated only scarcely. It was shown previously that ischemic injury leads to alterations in intracellular lipids, associated with changed expression of HMGCR [16, 27–30]. Also other renal injury processes are associated with altered renal lipid homeostasis [30]. These responses are considered primarily protective, and protect injured cells from additional injury. This earlier research focused on glomeruli and tubular cells. The regulation in renal vascular cells is less clear. Given the fact that the current study used core renal biopsies for gene expression evaluation, the cellular origin of the differentially expressed cholesterol pathway genes remains unclear.

Intimal hyperplasia is the universal response of a vessel to injury, like repeated hemodynamic and oxidative stress on the endothelium [31, 32]. Injury-induced intimal hyperplasia underlies the pathogenesis of major cardiovascular diseases [33]. The increased rate of cell turnover in regions with hemodynamic stress has been associated with accelerated telomere attrition and with endothelial cell senescence, which further contributes to endothelial dysfunction and intimal hyperplasia [7, 34–36]. Moreover, the secretory phenotype of senescent cells causes a full range of autocrine and paracrine activities, aimed at tissue repair, but also fuels degenerative and proliferative changes contributing to arteriosclerosis [9].

In the current study, we demonstrate for the first time that there are significant associations between renal arteriosclerosis, intrarenal telomere attrition and dysregulation of cholesterol biosynthesis in kidney biopsies. Notwithstanding the interest of this finding, the current study is not suited to distinguish causal relations between these factors. Telomere attrition and renal dysregulation of cholesterol metabolism could be caused by common risk factors that contribute to intrarenal arteriosclerosis. On the other hand, overexpression of the cholesterol biosynthesis genes could accelerate intrarenal arteriosclerosis and from this accelerated telomere attrition, as a protective mechanism [37]. Furthermore, it could be hypothesized that telomere attrition in itself leads to altered cholesterol biosynthesis and arteriosclerosis. Finally, it could be hypothesized that the observed alterations are rather protective in nature. Further evaluation of these very different hypotheses will be necessary to better understand the likely complex interplay between renal arteriosclerosis, senescence and cholesterol homeostasis. Given the suggestion that renal arteriosclerosis represents specific senescence processes, and the fact that cholesterol homeostasis can be influenced by relatively safe therapeutic interventions, our data-driven results provide first clues how progressive renal arteriosclerosis could be slowed down.

Our study has several limitations. It should be acknowledged that the associations between gene expression, telomere length and renal arteriosclerosis was not strong, and statistical analysis did not pass stringent Benjamini-Hochberg criteria for multiple testing. Nevertheless, the downstream pathway evaluation and independent validation of individual targets largely overcomes this drawback. Given this intrinsic drawback, it could be that this approach decreases the sensitivity to detect other significant genes or pathways associated with these phenotypes. Next, it should be emphasized that the primary micro-array analysis was performed on a low number of heterogeneous samples, especially with low prevalence of renal arteriosclerosis in the test cohort. Again, independent validation of the main findings decreases this issue, although the potential for type 2 errors remains. Another limitation of this study is that global intrarenal gene expression does neither provide any spatial information nor insight in the cellular origin of the detected signals. Given the apparent importance of cellular cholesterol homeostasis in our analysis, information on circulating and intrarenal cholesterol levels would be important for interpretation of our data, but these levels were not available. Although clinical demographics were collected prospectively, some kidney donor parameters that could be of importance could have been recorded incompletely. Finally, it has to be acknowledged that the reproducibility of semi-quantitative histologic scoring of kidney biopsies is moderate at best, and that sampling error is inherent to any biopsy study. Despite these shortcomings, the independent validation of the association between telomere length, renal arteriosclerosis and the cholesterol pathways supports the robustness and reproducibility of our data.

In conclusion, our study suggests that there is an association between overexpression of intrarenal cholesterol biosynthesis, shorter intrarenal telomere length and increased renal arteriosclerosis in humans, although no causality can be inferred from our data. Further studies are necessary to elucidate the cell types involved, the impact of circulating cholesterol levels and the causal relations. Whether treatment aimed at inhibiting cholesterol biosynthesis has beneficial effects on renal biological aging or renal arteriosclerosis, warrants further study.

## MATERIALS AND METHODS

### Inclusion and exclusion criteria

A test cohort of 40 consecutive kidneys, for transplantation in adult recipients between February 2013 and September 2013 at the University Hospital Leuven (Leuven, Belgium), was included in this study. Pre-implantation biopsies were performed in these kidneys, for evaluation of telomere length, gene expression and histological evaluation available. The validation cohort included kidney biopsies from 173 consecutive kidney donors for transplantation in adult recipients, transplanted between September 2013 and April 2015 at this same institution. This study was approved by the Ethics Committee/Institutional Review Board of the University Hospitals Leuven, Leuven, Belgium (OG032; ML7499 and ML9785; clinicaltrials.gov NCT01331668).

### Clinical data collection

Clinical data were obtained from the Eurotransplant database (“Eurotransplant Donor Report”), which is maintained prospectively and is the central source of donor data for organ transplantation in the Eurotransplant region. The following data were collected: calendar age, gender, cause of death, weight and length, living vs. deceased donor, brain death vs. cardiac death donor, body mass index, history of cardiovascular events prior to donation (including reason for death in deceased donors) and terminal serum creatinine levels before organ recovery. Renal function was estimated by the 4-variable Modification of Diet in Renal Disease (MDRD) equation (estimated glomerular filtration rate; eGFR) [39].

### Kidney biopsies and histologic evaluation

One pathologist (EL) reviewed all pre-implantation kidney biopsies, without knowledge of any demographic information. The biopsy specimens were wedge biopsies with slides containing 4 to 10 paraffin sections (2 μm) that were stained with hematoxylin eosin, periodic acid–Schiff, and a silver methenamine staining method (Jones). The severity of histologic lesions, interstitial fibrosis (Banff “ci” score), tubular atrophy (Banff “ct” score), arteriolar hyalinosis (Banff “ah” score) and arteriosclerosis (Banff “cv” score = vascular fibrous intimal thickening = intimal hyperplasia), were scored semiquantitatively according to the Banff criteria [40]. In addition, the total number of glomeruli in each biopsy, and the number of globally sclerosed glomeruli, were calculated separately. Only biopsies with >10 glomeruli (A quality) were included for evaluation of glomerulosclerosis.

### Telomere length in kidney biopsies

Since November 2012, next to the wedge biopsy that was used for histological evaluation, a full renal cortical biopsy core was obtained prior to implantation, and immediately stored in Allprotect Tissue Reagent (Qiagen, Venlo, The Netherlands) solution, until extraction.

DNA extraction was performed by the Allprep DNA/RNA/miRNA Universal Kit (Qiagen, Venlo, The Netherlands) on a QIAcube instrument (Qiagen, Venlo, The Netherlands). Both DNA yield (ng/μL) and purity ratios A260/280 and A260/230 were determined using a Nanodrop ND-1000 spectrophotometer (Isogen Life Science, De Meern, the Netherlands) and needed to be within strict quality limits (yield 50 ng/μL; purity ratio range 1.5-2 and 1.5-2 for A260/280 and A260/230, respectively) for inclusion of the samples in the study. Extracted DNA samples were stored at -80°C until further use.

Telomere length in renal tissue samples was measured based on a modified quantitative real-time PCR protocol [41]. Telomere lengths were expressed as the telomere repeat copy number relative to a single-copy gene (*36B4*). DNA samples were diluted to 5ng and checked using the Quant-it PicoGreen dsDNA assay kit (Life Technologies) to ensure uniform DNA input for PCR quantification. The telomere reaction mixture contained 1x Qiagen QuantiTect SYBR Green Mastermix, 2.5 mM of dithiothreitol, 300 nM of telg primer (5’-ACACTAAGGTTTGGGTTTGGGTTT- GGGTTTGGGTTAGTGT-3’), and 900 nM of telc primer (5’- TGTTAGGTATCCC- TATCCCTATCCCTATCCCTATCCCTAACA-3’). Telomere PCR conditions were: 1 cycle at 95°C for 10 min, followed by 2 cycles of 15 sec at 94°C and 2 min at 49°C, and 30 cycles of 15 sec at 94°C, 20 sec at 62°C and 1 min 40 sec at 74°C. The single-copy gene (36B4) reaction mixture contained 1x Qiagen QuantiTect SYBR Green Mastermix, 300nM 36B4U primer (5’-CAGCAAGTGGGAAGGTGTAATCC-3’) and 500nM 36B4D primer (5’-CCCATTCTATCATCAACGGGTACAA-3’). Single-copy gene PCR conditions were: 1 cycle at 95°C for 10 min, followed by 40 cycles at 95°C for 15 sec, and 58°C for 1 min 10 sec. Samples were run in triplicate on an Applied Biosystems 7900HT Fast Real-Time PCR system in a 384-well format. PCR efficiency was calculated based on a 6-point serial dilution (20ng-0.08ng) of pooled buffy-coat DNA and was accepted between limits of 90-110%. Relative average telomere lengths were calculated using qBase software (Biogazelle, Zwijnaarde, Belgium) and expressed as the ratio of telomere copy number to single-copy gene number (T/S) compared to the average T/S ratio of the entire population. We achieved a coefficient of variation within telomere and single-copy gene triplicates of 0.70% and 0.51% respectively. All biopsies passed quality control for assessment of intra-renal telomere length.

### Whole genome micro array mRNA expression analysis

In the test cohort (N=40), whole genome microarray mRNA expression analysis was performed using Affymetrix Gene 2.0 ST arrays (N=40), which covers in total 40.716 RefSeq transcripts and 11.086 lincRNA transcripts. Total RNA (150 ng) was used to analyse the mRNA expression via human Gene 2.0 ST arrays according to manufacturer’s manual (4475209 Rev.B; Applied Biosystems, CA and 702808 Rev.6; Affymetrix, CA). Briefly, in the first cycle, double stranded cDNA was prepared with random hexamers tagged with a T7 promoter sequence followed by the generation of cRNA using the GeneChip WT Synthesis and Amplification kit (Applied Biosystems, CA). cRNA concentration after cleanup was measured with the NanoDrop ND-1000 spectrophotometer (NanoDrop Technologies). In the second cycle, sense oriented single-stranded DNA containing dUTP was generated and the concentration was, after cleanup, measured using the NanoDrop spectrophotometer. cRNA was hydrolysed and the single-stranded DNA was fragmented using uracil DNA glycosylase (UDG) and apurinic/apyrimidinic endonuclease 1 (APE1) (GeneChip WT terminal Labeling kit, Affymetrix). The quality of fragmentation (fragments should be between 40 and 70 nucleotides) was checked on the Bioanalyzer (Agilent, Waldbronn, Germany). The fragmented DNA was labeled by terminal deoxynucleotidyl transferase (TDT) with the Affymetrix DNA Labeling reagent that is covalently linked to biotin (GeneChip WT terminal Labeling kit, Affymetrix). Labeled DNA was hybridized to the array during 17h at 45°C. The arrays were washed and stained in a fluidics station using the GeneChip hybridization, Wash end Stain kit (Affymetrix) and scanned using the Affymetrix 3000 GeneScanner. All image files were generated using the Affymetrix GeneChip command console (AGCC). The raw data were analysed with RMA sketch using the standard settings for Gene 2.0 ST arrays of Expression Console in the AGCC software.

### mRNA gene expression analysis

RT-PCR was performed in the validation cohort using OpenArray™ technology, a real-time PCR–based solution for high-throughput gene expression analysis (Quantstudio 12K Flex Real-Time PCR system, Thermofischer Scientific; Ghent, Belgium). After analysis of the intersecting pathways enriched in the micro-array gene expression analyses, expression of individual genes from the most robust pathway was validated. 14 genes were selected from the micro-array model (FDPS; DHCR7; LSS; HMGCR; FDFT1; IDI1; ACAT2; NSDHL; HMGCS1; CYP51A1; SQLE; MSMO1; MVD; HSD17B7) together with 3 housekeeping genes 18s, HPRT and GAPDH (Supplementary Table 5).

RNA extraction was performed by the Allprep DNA/RNA/miRNA Universal Kit (Qiagen, Venlo, The Netherlands) on a QIAcube instrument (Qiagen, Venlo, The Netherlands). cDNA synthesis was performed according the manufacturer’s instructions with 50 ng mRNA (Superscript VILO cDNA synthesis kit, Thermo Fisher Scientific, Gent, Belgium). Preamplification was performed according the manufacturer’s instructions. The pre-amplified cDNA was mixed with TaqMan Universal PCR Master Mix and injected on the OpenArray™ slide using OpenArray Accufill System (Thermo Fisher Scientific, Gent, Belgium). The OpenArray™ slides were spotted with the requested assays and the three endogenous controls 18S, HPRT and GAPDH by the manufacturer (Thermo Fisher Scientific, Gent, Belgium). Data were analyzed using the QuantStudio 12K flex software. Gene expression in each sample was calculated relative to the expression of a reference RNA sample, using the ΔΔCt method with log2 transformation.

### Statistical analysis

For the microarray gene expression analysis, multivariable linear regression was performed to examine the association between telomere length and log_2_-transformed gene expression level of each probe set, adjusted for calendar age, gender and batch number. Second, also multivariable logistic regression analysis was performed to examine the association between the presence of arteriosclerosis (Banff grade absent vs. present) and log_2_-transformed gene expression levels. Genes with a p-value smaller than 0.05 were classified as being significantly associated with telomere length or presence of arteriosclerosis. Also, Benjamini-Hochberg false discovery rate-adjusted p-value were calculated [42]. For biological interpretation, the differentially expressed genes, based on unadjusted p-values, were loaded into Qiagen’s Ingenuity® Pathway Analysis (IPA®) platform. Pathways with a q-value (Benjamini-Hochberg false discovery rate-adjusted p-value) below 0.05 were considered significantly overrepresented. In the validation cohort, gene expression differences between groups were analyzed using parametric or non-parametric one-way ANOVA. Associations between telomere length and gene expression were assessed by means of Spearman correlations. To integrate gene expression data for different transcripts, principal component analysis was used. From the principal component analysis, a principal component represents a linear combination of gene expression levels of a range of selected genes with as weights the eigenvectors. Principal components having eigenvalues >1 were selected. For variance analysis of continuous variables in different groups, non-parametric Wilcoxon-Mann-Whitney U, non-parametric ANOVA and parametric one-way ANOVA were used, as appropriate. Dichotomous variables were compared using the chi-square test. All tests were two-sided and p-values less than 0.05 were considered to indicate statistical significance. The results are expressed as numerical values and percentages for categorical variables and as mean ± standard deviation for continuous variables, unless stated otherwise. Analyses were done with SAS (version 9.2; SAS institute, Cary, NC), JMP9.0 (SAS institute, Cary, NC), GraphPad Prism (version 5.00; GraphPad Software, San Diego, CA) and Qiagen’s Ingenuity® Pathway Analysis (IPA®; Redwood City, CA).

## Supplementary Material

Supplementary Tables

## References

[1] Rodier F, Campisi J. Four faces of cellular senescence. J Cell Biol. 2011; 192:547–56. 10.1083/jcb.201009094 21321098 PMC3044123

[2] Calado RT, Young NS. Telomere diseases. N Engl J Med. 2009; 361:2353–65. 10.1056/NEJMra0903373 20007561 PMC3401586

[3] Hayflick L, Moorhead PS. The serial cultivation of human diploid cell strains. Exp Cell Res. 1961; 25:585–621. 10.1016/0014-4827(61)90192-6 13905658

[4] Wong JM, Collins K. Telomere maintenance and disease. Lancet. 2003; 362:983–88. 10.1016/S0140-6736(03)14369-3 14511933

[5] Nawrot TS, Staessen JA, Gardner JP, Aviv A. Telomere length and possible link to X chromosome. Lancet. 2004; 363:507–10. 10.1016/S0140-6736(04)15535-9 14975611

[6] De Vusser K, Pieters N, Janssen B, Lerut E, Kuypers D, Jochmans I, Monbaliu D, Pirenne J, Nawrot T, Naesens M. Telomere length, cardiovascular risk and arteriosclerosis in human kidneys: an observational cohort study. Aging (Albany NY). 2015; 7:766–75. 10.18632/aging.100814 26539975 PMC4637205

[7] Fuster JJ, Díez J, Andrés V. Telomere dysfunction in hypertension. J Hypertens. 2007; 25:2185–92. 10.1097/HJH.0b013e3282ef6196 17921808

[8] Fitzpatrick AL, Kronmal RA, Gardner JP, Psaty BM, Jenny NS, Tracy RP, Walston J, Kimura M, Aviv A. Leukocyte telomere length and cardiovascular disease in the cardiovascular health study. Am J Epidemiol. 2007; 165:14–21. 10.1093/aje/kwj346 17043079

[9] Fyhrquist F, Saijonmaa O, Strandberg T. The roles of senescence and telomere shortening in cardiovascular disease. Nat Rev Cardiol. 2013; 10:274–83. 10.1038/nrcardio.2013.30 23478256

[10] Rubin R. Rubin's Pathology: Clinicopathologic Foundations of Medicine. 2011. Lippincott Williams & Wilkins.

[11] Insull W Jr. The pathology of atherosclerosis: plaque development and plaque responses to medical treatment. Am J Med. 2009 (1 Suppl); 122:S3–14. 10.1016/j.amjmed.2008.10.013 19110086

[12] Rader DJ, Daugherty A. Translating molecular discoveries into new therapies for atherosclerosis. Nature. 2008; 451:904–13. 10.1038/nature06796 18288179

[13] Tedgui A, Mallat Z. Cytokines in atherosclerosis: pathogenic and regulatory pathways. Physiol Rev. 2006; 86:515–81. 10.1152/physrev.00024.2005 16601268

[14] Ross JS, Stagliano NE, Donovan MJ, Breitbart RE, Ginsburg GS. Atherosclerosis and cancer: common molecular pathways of disease development and progression. Ann N Y Acad Sci. 2001; 947:271–92. 10.1111/j.1749-6632.2001.tb03949.x 11795276

[15] Raskin P, Siperstein MD. Mevalonate metabolism by renal tissue in vitro. J Lipid Res. 1974; 15:20–25. 4811213

[16] Zager RA, Johnson AC, Hanson SY. Sepsis syndrome stimulates proximal tubule cholesterol synthesis and suppresses the SR-B1 cholesterol transporter. Kidney Int. 2003; 63:123–33. 10.1046/j.1523-1755.2003.00735.x 12472775

[17] Fornoni A, Merscher S, Kopp JB. Lipid biology of the podocyte—new perspectives offer new opportunities. Nat Rev Nephrol. 2014; 10:379–88. 10.1038/nrneph.2014.87 24861084 PMC4386893

[18] Hassan HH, Denis M, Krimbou L, Marcil M, Genest J. Cellular cholesterol homeostasis in vascular endothelial cells. Can J Cardiol. 2006; 22:35B–40B. 10.1016/s0828-282x(06)70985-0 16498511 PMC2780830

[19] Weber C, Noels H. Atherosclerosis: current pathogenesis and therapeutic options. Nat Med. 2011; 17:1410–22. 10.1038/nm.2538 22064431

[20] Pathway Unification Database. Pathcards.

[21] Brown MS, Goldstein JL. Familial hypercholesterolemia: defective binding of lipoproteins to cultured fibroblasts associated with impaired regulation of 3-hydroxy-3-methylglutaryl coenzyme A reductase activity. Proc Natl Acad Sci USA. 1974; 71:788–92. 10.1073/pnas.71.3.788 4362634 PMC388099

[22] Brown MS, Goldstein JL. The SREBP pathway: regulation of cholesterol metabolism by proteolysis of a membrane-bound transcription factor. Cell. 1997; 89:331–40. 10.1016/S0092-8674(00)80213-5 9150132

[23] Ren S, Ning Y. Sulfation of 25-hydroxycholesterol regulates lipid metabolism, inflammatory responses, and cell proliferation. Am J Physiol Endocrinol Metab. 2014; 306:E123–30. 10.1152/ajpendo.00552.2013 24302009 PMC3920008

[24] Reiss AB, Voloshyna I, De Leon J, Miyawaki N, Mattana J. Cholesterol Metabolism in CKD. Am J Kidney Dis. 2015; 66:1071–82. 10.1053/j.ajkd.2015.06.028 26337134 PMC4658227

[25] Karagiannis GS, Weile J, Bader GD, Minta J. Integrative pathway dissection of molecular mechanisms of moxLDL-induced vascular smooth muscle phenotype transformation. BMC Cardiovasc Disord. 2013; 13:4. 10.1186/1471-2261-13-4 23324130 PMC3556327

[26] Li H, Lewis A, Brodsky S, Rieger R, Iden C, Goligorsky MS. Homocysteine induces 3-hydroxy-3-methylglutaryl coenzyme a reductase in vascular endothelial cells: a mechanism for development of atherosclerosis? Circulation. 2002; 105:1037–43. 10.1161/hc0902.104713 11877351

[27] Zager RA, Johnson AC, Hanson SY, Shah VO. Acute tubular injury causes dysregulation of cellular cholesterol transport proteins. Am J Pathol. 2003; 163:313–20. 10.1016/S0002-9440(10)63655-3 12819036 PMC1868170

[28] Zager RA. Plasma membrane cholesterol: a critical determinant of cellular energetics and tubular resistance to attack. Kidney Int. 2000; 58:193–205. 10.1046/j.1523-1755.2000.00154.x 10886564

[29] Johnson AC, Ware LB, Himmelfarb J, Zager RA. HMG-CoA reductase activation and urinary pellet cholesterol elevations in acute kidney injury. Clin J Am Soc Nephrol. 2011; 6:2108–13. 10.2215/CJN.02440311 21799150 PMC3359009

[30] Abrass CK. Cellular lipid metabolism and the role of lipids in progressive renal disease. Am J Nephrol. 2004; 24:46–53. 10.1159/000075925 14707435

[31] Haycock PC, Heydon EE, Kaptoge S, Butterworth AS, Thompson A, Willeit P. Leucocyte telomere length and risk of cardiovascular disease: systematic review and meta-analysis. BMJ. 2014; 349:g4227. 10.1136/bmj.g4227 25006006 PMC4086028

[32] Bhayadia R, Schmidt BM, Melk A, Homme M. Senescence-Induced Oxidative Stress Causes Endothelial Dysfunction. J Gerontol A Biol Sci Med Sci. 2016; 71:161–9. 10.1093/gerona/glv008 25735595

[33] Griendling KK, FitzGerald GA. Oxidative stress and cardiovascular injury: Part II: animal and human studies. Circulation. 2003; 108:2034–40. 10.1161/01.CIR.0000093661.90582.c4 14581381

[34] Chang E, Harley CB. Telomere length and replicative aging in human vascular tissues. Proc Natl Acad Sci USA. 1995; 92:11190–94. 10.1073/pnas.92.24.11190 7479963 PMC40597

[35] Minamino T, Miyauchi H, Yoshida T, Ishida Y, Yoshida H, Komuro I. Endothelial cell senescence in human atherosclerosis: role of telomere in endothelial dysfunction. Circulation. 2002; 105:1541–44. 10.1161/01.CIR.0000013836.85741.17 11927518

[36] Matthews C, Gorenne I, Scott S, Figg N, Kirkpatrick P, Ritchie A, Goddard M, Bennett M. Vascular smooth muscle cells undergo telomere-based senescence in human atherosclerosis: effects of telomerase and oxidative stress. Circ Res. 2006; 99:156–64. 10.1161/01.RES.0000233315.38086.bc 16794190

[37] Muñoz-Espín D, Serrano M. Cellular senescence: from physiology to pathology. Nat Rev Mol Cell Biol. 2014; 15:482–96. 10.1038/nrm3823 24954210

[38] Levey AS, Coresh J, Greene T, Stevens LA, Zhang YL, Hendriksen S, Kusek JW, Van Lente F; Chronic Kidney Disease Epidemiology Collaboration. Using standardized serum creatinine values in the modification of diet in renal disease study equation for estimating glomerular filtration rate. Ann Intern Med. 2006; 145:247–54. 10.7326/0003-4819-145-4-200608150-00004 16908915

[39] Levey AS, Bosch JP, Lewis JB, Greene T, Rogers N, Roth D. A more accurate method to estimate glomerular filtration rate from serum creatinine: a new prediction equation. Modification of Diet in Renal Disease Study Group. Ann Intern Med. 1999; 130:461–70. 10.7326/0003-4819-130-6-199903160-00002 10075613

[40] Racusen LC, Solez K, Colvin RB, Bonsib SM, Castro MC, Cavallo T, Croker BP, Demetris AJ, Drachenberg CB, Fogo AB, Furness P, Gaber LW, Gibson IW, et al. The Banff 97 working classification of renal allograft pathology. Kidney Int. 1999; 55:713–23. 10.1046/j.1523-1755.1999.00299.x 9987096

[41] Cawthon RM. Telomere measurement by quantitative PCR. Nucleic Acids Res. 2002; 30:e47. 10.1093/nar/30.10.e47 12000852 PMC115301

[42] Hochberg Y, Benjamini Y. More powerful procedures for multiple significance testing. Stat Med. 1990; 9:811–18. 10.1002/sim.4780090710 2218183

