# Supplementary material for: Intrarenal arteriosclerosis and telomere attrition associate with dysregulation of the cholesterol pathway
Source: Aging (Albany NY). 2020 Apr 30;12(9):7830–47. doi: 10.18632/aging.103098 (PMC7244056; doi:10.18632/aging.103098)
Supplement: Supplementary Tables [file aging-12-103098-s001..pdf]

SUPPLEMENTARY TABLES

Supplementary Table 1. Significantly enriched pathways in association with telomere length and arteriosclerosis.

Ingenuity canonical pathways	Telomere model		Intrarenal arteriosclerosis model	
	q value	Ratio	q value	Ratio
Oxidative Phosphorylation	5.80E-10	2.45E-01	-	-
Superpathway of Cholesterol Biosynthesis	2.70E-08	4.44E-01	3.31E-04	3.70E-01
Mitochondrial Dysfunction	1.31E-06	1.56E-01	-	-
Cholesterol Biosynthesis I	1.31E-05	5.38E-01	0.21	3.08E-01
Cholesterol Biosynthesis II (via 24.25-dihydrolanosterol)	1.31E-05	5.38E-01	0.21	3.08E-01
Cholesterol Biosynthesis III (via Desmosterol)	1.31E-05	5.38E-01	0.21	3.08E-01
Superpathway of Geranylgeranyl diphosphate Biosynthesis I	0.01	3.12E-01	0.02	3.75E-01
Mevalonate Pathway I	0.04	3.33E-01	0.03	4.17E-01
Zymosterol Biosynthesis	0.04	5.00E-01	-	-

For biological interpretation, the significantly differentially expressed genes in the adjusted linear regression analysis for the telomere and intrarenal arteriosclerosis model respectively, were uploaded into Qiagen's Ingenuity® Pathway Analysis platform (IPA®; Redwood City. CA), to identify overrepresented pathways. Pathways with a q-value (false discovery rate adjusted p-value) below 0.05 were considered significantly overrepresented. The ratio expresses the log 2 fold change in pathway expression adjusted for the q value.

Supplementary Table 2. Significant genes in the 9 canonical pathways enriched in association with telomere length.

Symbol	Gene	Pathway			P value	Estimate°
ACAT2*	acetyl-CoA acetyltransferase 2	Superpathway of cholesterol biosynthesis	Superpathway of geranylgeranyldiphosphate	Mevalonate Pathway	0,01	-2.09
ATP5A1	ATP synthase, H+ transporting, mitochondrial F1 complex, alpha subunit 1	Oxidative Phosphorylation	Mitochondrial dysfunction		0.02	-0.59
ATP5G1	ATP synthase, H+ transporting, mitochondrial Fo complex, subunit C1 (subunit 9)	Oxidative Phosphorylation	Mitochondrial dysfunction		0.03	-0.90
ATP5I	ATP synthase, H+ transporting, mitochondrial Fo complex, subunit E	Oxidative Phosphorylation	Mitochondrial dysfunction		0.01	-0.84
ATP5L	ATP synthase, H+ transporting, mitochondrial Fo complex, subunit G	Oxidative Phosphorylation	Mitochondrial dysfunction		0.01	-1.09
ATP5S	ATP synthase, H+ transporting, mitochondrial Fo complex, subunit S	Oxidative Phosphorylation	Mitochondrial dysfunction		0.02	-0.90
COX4I2	cytochrome c oxidase subunit IV isoform 2	Oxidative Phosphorylation	Mitochondrial dysfunction		0.01	-1.03
COX5B	cytochrome c oxidase subunit Vb	Oxidative Phosphorylation	Mitochondrial dysfunction		0.006	-1.23
COX6B1	cytochrome c oxidase subunit VIb polypeptide 1	Oxidative Phosphorylation	Mitochondrial dysfunction		0.01	-1.09
COX7B	cytochrome c oxidase subunit VIIb	Oxidative Phosphorylation	Mitochondrial dysfunction		0.01	-0.87
CYC1	cytochrome c-1	Oxidative Phosphorylation	Mitochondrial dysfunction		0.02	-0.89
CYP51A1*	cytochrome P450, family 51, subfamily A, polypeptide 1	Superpathway of cholesterol biosynthesis	Superpathway of cholesterol biosynthesis		0.02	-1.38
DHCR7*	7-dehydrocholesterol reductase	Superpathway of cholesterol biosynthesis	Cholesterol biosynthesis I-II-III		0.006	-2.37
FDFT1*	farnesyl-diphosphate farnesyltransferase 1	Superpathway of cholesterol biosynthesis	Cholesterol biosynthesis I-II-III		0.007	-1.24
FDPS*	farnesyl diphosphate synthase	Superpathway of cholesterol biosynthesis	Superpathway of geranylgeranyldiphosphate		0.004	-1.12
HMGCR*	3-hydroxy-3-methylglutaryl-CoA reductase	Superpathway of cholesterol biosynthesis	Superpathway of geranylgeranyldiphosphate	Mevalonate Pathway	0.007	-1.63
HMGCS1*	3-hydroxy-3-methylglutaryl-CoA synthase 1	Superpathway of cholesterol biosynthesis	Superpathway of geranylgeranyldiphosphate	Mevalonate Pathway	0.02	-1.97
IDI1*	isopentenyl-diphosphate delta isomerase 1	Superpathway of cholesterol biosynthesis	Superpathway of geranylgeranyldiphosphate	Mevalonate Pathway	0.01	-1.83
LSS*	lanosterol synthase (2,3-oxidosqualene-lanosterol cyclase)	Superpathway of cholesterol biosynthesis	Cholesterol biosynthesis I-II-III		0.006	-1.80
MSMO1*	methylsterol monooxygenase 1	Superpathway of cholesterol biosynthesis	Cholesterol biosynthesis I-II-III	Zymosterol biosynthesis	0.03	-1.97
NDUFA1	NADH dehydrogenase (ubiquinone) 1 alpha subcomplex, 1	Oxidative Phosphorylation	Mitochondrial dysfunction		0.02	-0.84
NDUFA10	NADH dehydrogenase (ubiquinone) 1 alpha subcomplex, 10	Oxidative Phosphorylation	Mitochondrial dysfunction		0.03	-0.53
NDUFA9	NADH dehydrogenase (ubiquinone) 1 alpha subcomplex, 9	Oxidative Phosphorylation	Mitochondrial dysfunction		0.04	-0.74
NDUFB1	NADH dehydrogenase (ubiquinone) 1 beta subcomplex, 1	Oxidative Phosphorylation	Mitochondrial dysfunction		0.02	-0.69
NDUFB8	NADH dehydrogenase (ubiquinone) 1 beta subcomplex, 8	Oxidative Phosphorylation	Mitochondrial dysfunction		0.01	-0.60

NDUFB9	NADH dehydrogenase (ubiquinone) 1 beta subcomplex, 9	Oxidative Phosphorylation	Mitochondrial dysfunction		0.01	-0.74
NDUFS1	NADH dehydrogenase (ubiquinone) Fe-S protein 1, (NADH-coenzyme Q reductase)	Oxidative Phosphorylation	Mitochondrial dysfunction		0.01	-0.90
NDUFS3	NADH dehydrogenase (ubiquinone) Fe-S protein 3, (NADH-coenzyme Q reductase)	Oxidative Phosphorylation	Mitochondrial dysfunction		0.02	-0.68
NDUFS7	NADH dehydrogenase (ubiquinone) Fe-S protein 7, (NADH-coenzyme Q reductase)	Oxidative Phosphorylation	Mitochondrial dysfunction		0.005	-0.96
NDUFS8	NADH dehydrogenase (ubiquinone) Fe-S protein 8, (NADH-coenzyme Q reductase)	Oxidative Phosphorylation	Mitochondrial dysfunction		0.004	-1.18
NSDHL*	NAD(P) dependent steroid dehydrogenase-like	Superpathway of cholesterol biosynthesis	Superpathway of cholesterol biosynthesis		0.01	-1.01
PINK1	PTEN Induced Putative Kinase 1	Mitochondrial dysfunction			0.01	-0.78
SQLE*	squalene epoxidase	Superpathway of cholesterol biosynthesis	Cholesterol biosynthesis I-II-III	Epoxysqualene biosynthesis	0.02	-2.18
UQCRC1	ubiquinol-cytochrome c reductase core protein I	Oxidative Phosphorylation	Mitochondrial dysfunction		0.007	-0.77
UQCRC2	ubiquinol-cytochrome c reductase core protein II	Oxidative Phosphorylation	Mitochondrial dysfunction		0.001	-1.09
VPS9D1	VPS9 domain containing 1	Oxidative Phosphorylation	Mitochondrial dysfunction		0.005	-0.91

Multiple linear regression for association of gene expression with telomere length, adjusted for calendar age, gender and microarray batch number, was used to model the determinants (gene expression) of telomere length. ° The estimate expresses the log2 fold change in gene expression for 1 standard deviation increase log T/S ratio. * Genes significant in test cohort selected for validation.

Supplementary Table 3. Significant genes in the 9 canonical pathways enriched in association with intrarenal arteriosclerosis.

Symbol	Gene	Pathway			P value	Estimate°
ACAT2*	acetyl-CoA acetyltransferase 2	Superpathway of cholesterol biosynthesis	Superpathway of geranylgeranyldiphosphate	Mevalonate Pathway	0.04	0.55
CPSF2	Cleavage And Polyadenylation Specific Factor 2	Cleavage and polyadenylation of pre RNA			0.04	-2.09
PAPOLA	Poly(A) Polymerase Alpha	Cleavage and polyadenylation of pre RNA			0.04	-1.09
FDFT1*	farnesyl-diphosphate farnesyltransferase 1	Superpathway of cholesterol biosynthesis	Cholesterol biosynthesis I-II-III		0.009	0.38
FDPS*	farnesyl-diphosphate farnesyltransferase 1	Superpathway of cholesterol biosynthesis	Superpathway of geranylgeranyldiphosphate	Trans, trans-farnesyl diphosphate biosynthesis	0.02	0.26
HMGCR*	3-hydroxy-3-methylglutaryl-CoA reductase	Superpathway of cholesterol biosynthesis	Superpathway of geranylgeranyldiphosphate	Mevalonate Pathway	0.02	0.44
HMGCS1 *	3-hydroxy-3-methylglutaryl-CoA synthase 1	Superpathway of cholesterol biosynthesis	Superpathway of geranylgeranyldiphosphate	Mevalonate Pathway	0.04	0.75
IDI1*	isopentenyl-diphosphate delta isomerase 1	Superpathway of cholesterol biosynthesis	Superpathway of geranylgeranyldiphosphate	Mevalonate Pathway	0.02	0.56
LSS*	lanosterol synthase (2,3-oxidosqualene-lanosterol cyclase)	Superpathway of cholesterol biosynthesis	Cholesterol biosynthesis I-II-III		0.02	0.47
MSMO1*	methylsterol monooxygenase 1	Superpathway of cholesterol biosynthesis	Cholesterol biosynthesis I-II-III	Zymosterol biosynthesis	0.03	-1.98
NSDHL*	NAD(P) dependent steroid dehydrogenase-like	Superpathway of cholesterol biosynthesis	Cholesterol biosynthesis I-II-III		0.01	-1.01
SQLE*	squalene epoxidase	Superpathway of cholesterol biosynthesis	Cholesterol biosynthesis I-II-III	Epoxysqualene biosynthesis	0.02	0.67
MVD*	mevalonate (diphospho) decarboxylase	Superpathway of cholesterol biosynthesis	Mevalonate Pathway	Superpathway of geranylgeranyldiphosphate	0.04	0.3
HSD17B7 *	hydroxysteroid (17-beta) dehydrogenase 7	Superpathway of cholesterol biosynthesis	Cholesterol biosynthesis I-II-III		0.02	0.55

Multiple linear regression adjusted for calendar age, gender and microarray batch number, was used to model the determinants (gene expression) of intrarenal arteriosclerosis. ° The estimate expresses the log2 fold change in gene expression for 1 standard deviation increase in presence of arteriosclerosis. * Gene expression validated in validation set.

Supplementary Table 4. Relation between intrarenal arteriosclerosis and donor history of cardiovascular events in the validation cohort and gene expression.

Intra renal arteriosclerosis				Donor history of cardiovascular events			
Symbol	Gene expression without arteriosclerosis	Gene expression with arteriosclerosis	P value	Symbol	Donor history of cardiovascular events absent	Donor history of cardiovascular events present	P value
SQLE	2.41 ± 0.10	3.24 ± 0.5	p=0.01	SQLE	2.16 ± 0.1	2.93 ± 0.19	p=0.0004
FDPS	1.22 ± 0.02	1.39 ± 0.11	p=0.04	FDPS	1.21 ± 0.03	1.32 ± 0.05	p=0.03
MVD	1.42 ± 0.07	1.60 ± 0.04	p=0.02	MVD	1.41 ± 0.04	1.68 ± 0.06	p=0.0003
HMGCS1	1.83 ± 0.07	2.30 ± 0.33	p=0.05	HMGCS1	1.74 ± 0.09	2.16 ± 0.16	p=0.009
HSD17B7	2.30 ± 0.10	5.86 ± 3.52	p=0.004	FDFT	1.21 ± 0.04	1.49 ± 0.06	p=0.0004
HSF1	0.88 ± 0.05	1.89 ± 1.02	p=0.007	CYP51A1	1.52 ± 0.05	1.77 ± 0.07	p=0.003
				DHCR7	1.88 ± 0.09	2.29 ± 0.12	p=0.005
				IDI1	0.93 ± 0.03	1.05 ± 0.04	p=0.01
				LSS	1.28 ± 0.05	1.44 ± 0.06	p=0.04
				MSMO1	2.28 ± 0.1	2.77 ± 0.17	p=0.01
				NSDL	1.16 ± 0.03	1.36 ± 0.05	p=0.0008

The p-values represent non-parametric ANOVA.

Supplementary Table 5. List of the genes evaluated using TaqMan gene expression assays.

Symbol	Entrez gene name	Lifetech array nr
FDPS	farnesyl diphosphate synthase	Hs01578769_g1
DHCR7	7-dehydrocholesterol reductase	Hs01023087_m1
LSS	lanosterol synthase (2.3-oxidosqualene-lanosterol cyclase)	Hs00158906_m1
HMGCR	3-hydroxy-3-methylglutaryl-CoA reductase	Hs00168352_m1
FDFT1	farnesyl-diphosphate farnesyltransferase 1	Hs00926054_m1
IDI1	isopentenyl-diphosphate delta isomerase 1	Hs01057440_m1
ACAT2	acetyl-CoA acetyltransferase 2	Hs00255067_m1
NSDHL	NAD(P) dependent steroid dehydrogenase-like	Hs00210873_m1
HMGCS1	3-hydroxy-3-methylglutaryl-CoA synthase 1 (soluble)	Hs00940429_m1
CYP51A1	cytochrome P450, family 51, subfamily A, polypeptide 1	Hs01567880_g1
SQLE	squalene epoxidase	Hs01123768_m1
MSMO1	methylsterol monooxygenase 1	Hs00932159_m1
MVD	mevalonate (diphospho) decarboxylase	Hs00159403_m1
HSD17B7	hydroxysteroid (17-beta) dehydrogenase 7	Hs00367686_m1
